# Who Shall Teach the Teachers?

**Published:** 1886-05

**Authors:** 


### "WHO SHALL TEACH THE TEACHERS?"

The *New York Medical Record* does not know "whether or not the *Lancet* is trying to poke fun at some of our eminent professors"—"if not, it shows that it possesses a remarkably chaotic notion of American surgical work."

The *Record*, commenting thus on an editorial in the *Lancet*, on "Laparotomy in gunshot wounds," in which credit is given Dr. F. S. Dennis for "the most important result of the labors of American surgeons in this line since the death of Garfield" (a paper by Dr. Dennis), goes on to read the *Lancet* a lesson, as follows:

"It is very well known to Americans surgeons that it was the cases of Dr. Wm. T. Bull and subsequently those [sic] of Dr. J. B. Hamilton and others, which especially turned the attention in this country to laparotomy in abdominal wounds. This Dr. Dennis himself acknowledges." (*Record* May 8.)

We were under the impression that it was "very well known to American surgeons" that the labors of J. Marion Sims, in the Franco-Prussian war, and subsequently in America; the admirable paper by Hunter McQuire, read before the American Medican Association some years ago, and the writings, and experiments on dogs, by Prof. Chas T. Parke of Chicago, "which especially turned the attention in this country" to the subject, and which induced Drs. Bull, Hamilton and others to undertake the operation. The record is that Dr. Bull performed the first *two* successful operations on the human subject, and Dr. Hamilton the third *one*; and Dr. Parke's success in the operation on dogs had much to do, no doubt with "turning their attention" to the subject, as well as Dr. McGuire's paper, in which he boldly advocated the cutting down upon the wound—tying bleeding vessels—removing all extravasated blood and all extraneous matter, etc., long anterior to Dr. Bull's case. This, we believe, Drs. Bull and Hamilton, and even "Dr. Dennis himself, will acknowledge."

We fear our learned contemporay of the *Record* is a little "chaotic" in the matter of "laparotomy in gunshot wounds."

